# Further evidence of involvement of *TMEM132E* in autosomal recessive nonsyndromic hearing impairment

**DOI:** 10.1038/s10038-019-0691-4

**Published:** 2019-10-28

**Authors:** Khurram Liaqat, Shabir Hussain, Muhammad Bilal, Abdul Nasir, Anushree Acharya, Raja Hussain Ali, Shoaib Nawaz, Muhammad Umair, Isabelle Schrauwen, Wasim Ahmad, Suzanne M. Leal

**Affiliations:** 1 Department of Biotechnology, Faculty of Biological Sciences, Quaid-i-Azam University, Islamabad, Pakistan; 2 Center of Statistical Genetics, Department of Molecular and Human Genetics, Baylor College of Medicine, Houston, TX USA; 3 Department of Biochemistry, Faculty of Biological Sciences, Quaid-i-Azam University, Islamabad, Pakistan; 4 Department of Molecular Science and Technology, Ajou University, Suwon, 443-749 South Korea; 5 Center for Statistical Genetics, Gertrude H. Sergievsky Center, Taub Institute for Alzheimer’s Disease and the Aging Brain, Department of Neurology, Columbia University Medical Center, 630 W 168th St, New York, NY 10032 USA; 6 Division of Hematology/Oncology, Boston Children’s Hospital, Boston, MA 02115 USA; 7 Medical Genomics Research Department, King Abdullah International Medical Research Center (KAIMRC), King Saud bin Abdulaziz University for Health Science, Ministry of National Guard-Health Affairs (MNGHA), P.O. Box 3660, Riyadh, 11481 Saudi Arabia

**Keywords:** Medical genomics, Next-generation sequencing

## Abstract

Autosomal-recessive (AR) nonsyndromic hearing impairment (NSHI) displays a high degree of genetic heterogeneity with >100 genes identified. Recently, *TMEM132E*, which is highly expressed in inner hair cells, was suggested as a novel ARNSHI gene for DFNB99. A missense variant c.1259G>A: p.(Arg420Gln) in *TMEM132E* was identified that segregated with ARNSHI in a single Chinese family with two affected members. In the present study, a family of Pakistani origin with prelingual profound sensorineural hearing impairment displaying AR mode of inheritance was investigated via exome and Sanger sequencing. Compound heterozygous variants c.382G>T: p.(Ala128Ser) and c.2204C>T: p.(Pro735Leu) in *TMEM132E* were observed in affected but not in unaffected family members. TMEM*132E* variants identified in this and the previously reported ARNSHI family are located in the extracellular domain. In conclusion, we present a second ARNSHI family with *TMEM132E* variants which strengthens the evidence of the involvement of this gene in the etiology of ARNSHI.

## Introduction

Hearing impairment (HI) is a heterogeneous disorder that occurs at all ages with varying severity, affecting 1 in 500 newborns and >360 million people worldwide [1]. To date, more than 100 autosomal recessive nonsyndromic hearing impairment (NSHI) genes have been identified. Due to extreme locus heterogeneity and the very low frequency of many ARNSHI variants often candidate HI genes have only been observed in a single family [1]. Identification of additional families is important to confirm their role in disease pathogenesis and to improve genotype–phenotype correlation.

The TMEM132 family contains five genes *TMEM132A, TMEM132B, TMEM132C, TMEM132D*, and *TMEM132E*. The role of the TMEM132 gene family remains poorly understood, however a few studies have reported their involvement in NSHI, panic disorders, and cancer [2].

*TMEM132E* (MIM 616178) is located within the DFNB99 locus on chromosome 17q12. This gene contains ten exons and encodes the TMEM132E protein which is highly expressed in the inner ear and other tissues including brain, kidney, lung, liver, spleen, heart, small intestine, colon, thymus, and stomach. In 2015, *TMEM132E* was suggested as a causative gene for ARNSHI [3, 4]. The main function of the TMEM132E protein is to connect the extracellular medium with the intracellular actin cytoskeleton [2].

To date only one *TMEM132E* variant has been suggested to be associated with ARNSHI [4]. We describe a family with *TMEM132E* compound heterozygous variants that segregate with ARNSHI, adding support of the involvement of this gene in HI etiology.

## Methods

### Clinical and molecular evaluation

**Fig. 1:**
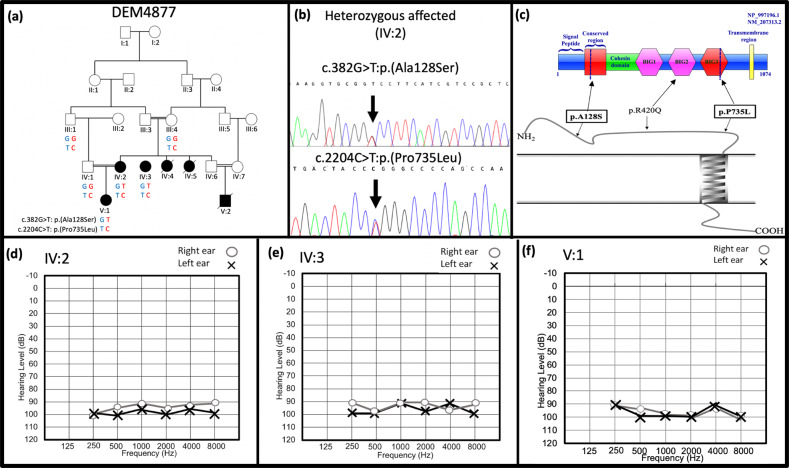
Pedigree diagram, sequence chromatogram, and audiograms of family DEM4877. **a** DEM4877 pedigree drawings and segregations results for T*MEM132E* variants c.382G>T: p.(Ala128Ser) and c.2204C>T: p.(Pro735Leu). Squares represent males and circles females; filled symbols denote hearing impaired individuals and clear symbols unaffected family members. Double lines indicate consanguineous marriages. **b** Sequencing chromatogram of *TMEM132E* compound heterozygous variants c.382G>T: p.(Ala128Ser) (upper panel) and c.2204C>T: p.(Pro735Leu) (lower panel) of affected individual IV:2 from family DEM4877. **c** The domain architecture of the TMEM132 protein family (predicted by Sanchez-Pulido and Chris P. Ponting, 2018) (upper panel) and predicted transmembrane helices in TMEM132E (adapted from the result of TMHMM 2.0 analysis) (lower panel) showing the positions of previously reported one missense p.(Arg420Gln) variant mapped on BIG2 domain, a novel variants p.(Ala128Ser) located in the conserved region (CR) and another variant p.(Pro735Leu) lies in the BIG3 domain. The previously reported and new ARNSHI variants located in the extracellular region of TMEM132E. Variants in the box were identified in this study. **d**–**f** Displays the pure tone audiograms (bone conduction audiometry can be found in Supplementary Fig. 1) for affected individuals **d** IV:2, **e** IV:3, and **f** V:1, respectively

This study was approved by the Institutional Review Boards of the Quaid-i-Azam University and the Baylor College of Medicine and Affiliated Hospitals. Consanguineous family, DEM4877, with ARNSHI was ascertained from a rural area of Sindh Province of Pakistan. Written informed consent was obtained from all participating members of DEM4877 and peripheral blood samples were collected from six family members: IV:2, IV:3, and V:1 who are affected with sensorineural HI and III:1, III:4, and IV:1 who are unaffected (Fig. 1a). Extraction of genomic DNA was performed using a Phenol–chloroform method [5]. Hearing impaired family members underwent pure tone air and bone conduction audiometry at a local government hospital (Fig. 1d–f and Supplementary Fig. 1). Tandem gait and Romberg tests were also performed to exclude vestibular dysfunction.

### Initial screening of *GJB2*, *SLC26A4*, *HGF*, and *CIB2*

Prior to exome sequencing, the entire coding region of *GJB2*, which is a common cause of HI, was screened by Sanger sequencing. Additional common ARHI variants in the Pakistani population were also screened via Sanger sequencing: i.e., intronic *HGF* variants (c.482+1986_1988delTGA and c.482+1991_2000delGATGATGAAA), missense variants p.(Phe91Ser), and p.(Cys99Trp) within *CIB2* and *SLC26A4* missense variants p.(Gln446Arg) and p.(Val239Asp) [6–8].

### Exome sequencing and bioinformatics analysis

Exome sequencing was performed using a DNA sample from affected DEM4877 pedigree member IV:3. For the preparation of exome libraries, the SureSelect Human All Exon V6 kit was used, that target 60.46 Mb of coding sequences in the human genome (~20,000 protein coding genes), which is 99% of the protein coding regions of the National Center for Biotechnology Information‘s reference sequence (RefSeq) database [9], the consensus coding sequence (CCDS) [10] project and GENCODE [11]. Sequencing was performed using 100 bp paired-end on a HiSeq2500/4000 instrument (Illumina Inc, San Diego, CA, USA). The mean sequencing depth for targeted regions is 64.22×. Reads were aligned to the Human genome (hg19/GRC37) using the Burrows–Wheeler algorithm and duplicates were removed with Picard (GATKIndelRealigner). Single-nucleotides variants and small insertions/deletions (Indels) were called using GATK as described previously [12]. Conservation and damaging effects of the variants were evaluated in silico using annotation tools incorporated in dbNSFP and ANNOVAR [13, 14]. Filtering was performed to analyze the exome sequence data. Frameshift, in-frame indels, missense with a Combined Annotation-Dependent Depletion (CADD) C-score >15, start/stop altering, nonsense, and splice-site variants with a minor allele frequency <0.005 in every Genome Aggregation Database (gnomAD) [15] population were retained. Segregation of the identified variants in the family was validated by Sanger sequencing using the BigDye terminator v3.1 on an ABI 3130 Genetic Analyzer (Applied Biosystems, Foster City, CA) (Fig. 1b). Primers surrounding region of interest were designed using primer3 software [16].

### Three-dimensional modeling

The transmembrane helical structure was predicted using TMHMM server v. 2.0 [17]. The three-dimensional structure of TMEM132E was built using the I-TASSER server based on ab initio/threading method [18]. For evaluation of stereochemical quality of protein structure, the PROCHECK program was used [19].

## Results

### Clinical description

Pure tone air and bone conduction audiometry diagnosed bilateral profound sensorineural HI in all DEM4877 affected pedigree members IV:2, IV:3, and V:1 (Fig. 1d–f and Supplementary Fig. 1). HI is prelingual and most likely congenital, since it was detected before the age of one in all affected family members. The hearing impaired family members were physically examined, neither gross vestibular dysfunction nor any episodes of vertigo were observed.

### Whole exome and Sanger sequencing

*GJB2* as well as selected variants in *CIB2*, HGF, and *SLC26A4* were excluded as the underlying cause of HI in family DEM4877. Exome sequencing was performed using a DNA sample from individual IV:3. After variant filtering to identify homozygous and potentially compound heterozygous rare variants, four homozygous variants in *ERICH3, DNAH11, SNAPC4*, and *RIMBP2* and potential compound heterozygous variants in *PHLDB1* and *TMEM132E* were observed and tested for segregation (Supplementary Table 1), No rare homozygous variants were found in the previously reported HI genes. Sanger sequencing confirmed the segregation of the compound heterozygous variants c.382G>T: p.(Ala128Ser) and c.2204C>T: p.(Pro735Leu) in *TMEM132E* (ENSG00000181291) (Fig. 1b). The compound variants in *PHLDB1* as well as homozygous variants in *ERICH3, DNAH11, SNAPC4*, and *RIMBP2* did not segregate with ARNSHI.

### In silico analysis

Both *TMEM132E* variants c.382G>T: p.(Ala128Ser) and c.2204C>T: p.(Pro735Leu) have high CADD C-scores of 24.3 and 23, respectively and are also predicted to be damaging by various bioinformatic tools (Supplementary Table 1). The variants are both rare in gnomAD (Supplementary table 1) with no homozygous variants observed.

### Three-dimensional modeling

**Fig. 2:**
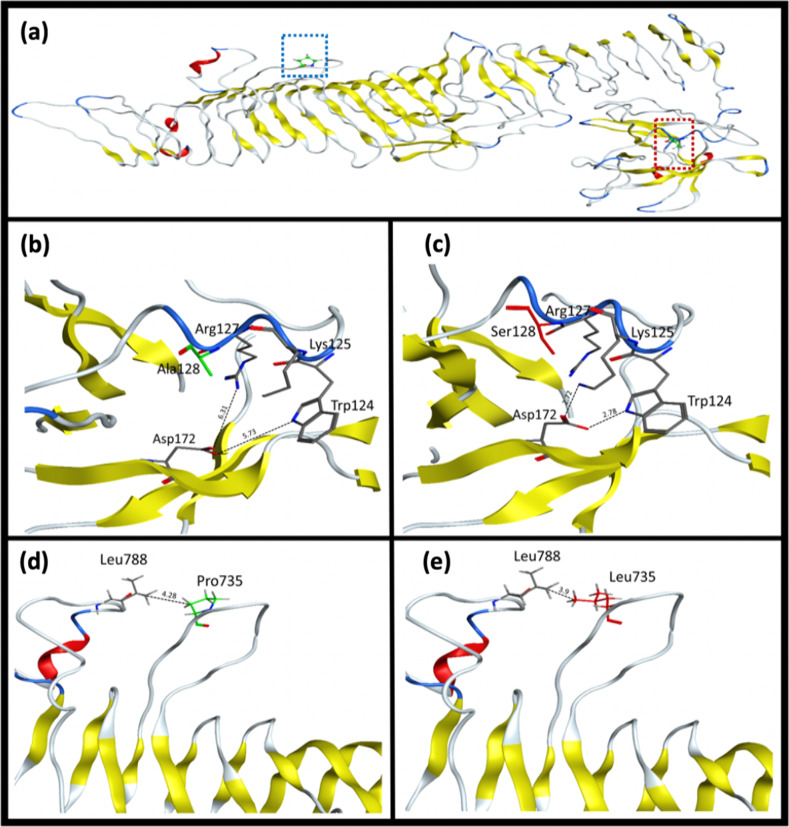
Predicted three-dimensional structure of TMEM132E. **a** Ball and stick model representation of p.Ala128 and p.Pro735 residues were highlighted by red and blue square respectively while **b**–**e** showing the interaction pattern of amino acid residues. **b** p.Ala128 (wild type), **c** p.Ser128 (mutant), **d** p.Pro735 (wild type) and **e** p.Leu735 (mutant)

TMHMM analysis demonstrated that both identified variants lie in the extracellular domain of the TMEM132E protein (Fig. 1c). In addition, we carried out comparative modeling to determine the structural difference between the wild type and mutant TMEM132E structure. Both the p.Ala128 and p.Pro735 positions lie in the loop region of the extracellular surface region of the protein. Residue p.Ala128 is not involved in any interaction with nearby residues, while p.Pro735 shows a hydrophobic interaction with a nearby p.Leu788 residue. In the case of the p.Ala128Ser variant, the insertion of polar charged side chain of serine residue establishes a strong interaction network with the nearby residues while the p.Pro735Leu variant shows local conformational changes in nearby residues (Fig. 2a–e).

## Discussion

In this study, we investigated three family members (IV:2, IV:3, and V:1) with prelingual profound HI and three unaffected members (II:1, II:4, and IV:1) of a consanguineous family. Neither vestibular dysfunction nor any other abnormality was observed in the affected individuals, suggesting these patients present with NSHI. Analysis of exome sequence data obtained from hearing impaired family member IV:3 revealed potentially compound heterozygous variants c.382G>T: p.(Ala128Ser) and c.2204C>T: p.(Pro735Leu) in *TMEM132E*. Sanger sequencing of the remaining family members confirmed the compound heterozygous status and demonstrated that these variants segregate with HI in DEM4877. To date, only one homozygous missense variant c.1259G>A: p.(Arg420Gln) in *TMEM132E* has been reported in a Chinese family segregating ARNSHI, suggesting involvement of the gene in HI [2, 4]. Through three-dimensional modeling of TMEM132E, we show that the variants p.(Ala128Ser) and p.(Pro735Leu) which segregate with ARNSHI in family DEM4877 cause local conformational changes due to the insertion of a new interaction network (Fig. 2b–e). TMHMM analysis revealed that all three amino acid residues altered in ARNSHI, p.Ala128, p.Arg420, and p.Pro735, are located in the extracellular region of TMEM132E protein. The extracellular portions of TMEM132 proteins contain three important regions i.e., a conserved region (CR), a cohesion domain (Ch) and three bacterial immunoglobulin-like (BIG) domains BIG1, BIG2, and BIG3 [2] (Fig. 1c). The variants p.(Ala128Ser) and p.(Pro735Leu) identified in the present study are located in the CR and the BIG3 domain respectively, while the previously identified variant p.(Arg420Gln) lies in the extracellular BIG2 domain of TMEM132E protein [2] (Fig. 1c). Due to the location of all three variants, the extracellular region of TMEM132E is likely critically important for proper function of the protein.

The *TMEM132E* gene is highly expressed in the brain and involved in neuronal function. Furthermore, mouse model studies show that *Tmem132e* is highly expressed in the inner hair cells of the cochlea, apical, and basal region of outer hair-cell cytoplasm but not in the hair bundle, in mice organ of Corti [4]. Knockdown of its ortholog (tmem132e) in *Danio rerio* affects the mechanotransduction of hair cells [4].

In conclusion, we identified compound heterozygous variants c.382G>T: p.(Ala128Ser) and c.2204C>T: p.(Pro735Leu) in *TMEM132E* that segregates with HI in a consanguineous Pakistani family. The previously reported *TMEM132E* variant in a Chinese family and newly identified compound heterozygous variants in Pakistani family suggesting that this gene could be involved in HI in both east and south Asian populations. This finding provides additional evidence for the involvement of *TMEM132E* in ARNSHI and expands the spectrum of variants involved in HI in the Pakistani population.

### Web resources

ANNOVAR, http://annovar.openbioinformatics.org/

Burrows–Wheeler Aligner, http://bio-bwa.sourceforge.net/

Combined Annotation-Dependent Depletion (CADD), https://cadd.gs.washington.edu/

dbSNP, https://www.ncbi.nlm.nih.gov/projects/SNP/

Exome Aggregation Consortium (ExAC), http://exac.broadinstitute.org/

gnomAD, http://gnomad.broadinstitute.org/

Genome Analysis Toolkit (GATK), https://software.broadinstitute.org/gatk/

Genomic Evolutionary Rate Profiling (GERP), http://mendel.stanford.edu/SidowLab/downloads/gerp/

Hereditary hearing loss, https://hereditaryhearingloss.org/

MutationTaster, http://www.mutationtaster.org/

Online Mendelian Inheritance of Man (OMIM), https://www.omim.org/

PhastCons and PhyloP, http://compgen.cshl.edu/phast/

Picard, https://broadinstitute.github.io/picard/

PyMol, https://pymol.org/

eXome-Hidden Markov Model (XHMM), https://atgu.mgh.harvard.edu/xhmm/

Uniport, http://www.uniprot.org/

UCSF Chimera, http://www.rbvi.ucsf.edu/chimera

## Supplementary information

Supplementary data
